# Osteonecrosis of the Jaw in Myeloma Patients Receiving Denosumab or Zoledronic Acid. Comment on Pivotal Trial by Raje et al. Published on Lancet Oncology

**DOI:** 10.3390/dj6030042

**Published:** 2018-09-01

**Authors:** Vittorio Fusco, Giuseppina Campisi, Paul de Boissieu, Federico Monaco, Anna Baraldi, Gianmauro Numico, Alberto Bedogni

**Affiliations:** 1 Oncology Unit, Azienda Ospedaliera di Alessandria, 15121 Alessandria, Italy; gianmauro.numico@ospedale.al.it; 2 Oral Medicine Unit, Università di Palermo, 90127 Palermo, Italy; campisi@odonto.unipa.it; 3 Service D’épidémiologie et de Santé Publique, Hôpitaux Universitaires Paris-Sud, 94270 Paris, France; paul.de-boissieu@u-psud.fr; 4 Haematology Unit, Azienda Ospedaliera di Alessandria, 15121 Alessandria, Italy; monacofederico.ospedale@gmail.com (F.M.); abaraldi@ospedale.al.it (A.B.); 5 Regional Center for Prevention, Diagnosis and Treatment of Medication and Radiation-Related Bone Diseases of the Head and Neck, University of Padua, 35136 Padua, Italy; alberto.bedogni@unipd.it

**Keywords:** osteonecrosis of the jaw, ONJ, osteonecrosis, jaw, medication-related osteonecrosis of the jaw, MRONJ, multiple myeloma, denosumab, zoledronic acid

## Abstract

The recent randomized trial, published by Raje et al., on Lancet Oncology is potentially practice changing. It proposes that denosumab is a valid alternative to zoledronic acid in the treatment of myeloma patients. However, several points need further data and more details, such as information on incidence, diagnosis, and follow-up of osteonecrosis of the jaw (ONJ) cases, observed among treated patients. Adopted definition to adjudicate ONJ cases, type of registration of potential ONJ cases, length of observation are possible causes of potential underestimation of ONJ incidence in their study. Future updated evaluations with longer follow-up, and including actuarial estimation, are required for final judgment on ONJ risk in myeloma patients receiving denosumab, and comparison with ONJ risk by zoledronic acid.

## Comment

Raje et al. [1] have published an article, in the March 2018 issue of Lancet Oncology, which describes an important trial—potentially changing clinical practice in therapy of myeloma patients.

Multiple myeloma leads to skeletal-related events (SREs), spinal cord compression, pathological fracture, or surgery or radiotherapy to affected bone. Bisphosphonates (zoledronic acid, pamidronate, clodronate) are generally used in myeloma patients, to reduce the risk of SREs. Denosumab, a monoclonal antibody targeting receptor activator of nuclear factor kappa-Β ligand (RANKL), showed to reduce SREs associated with bone lesions or metastases in patients with advanced solid tumors. It was therefore investigated in myeloma patients.

The study by Raje et al. [1] aimed to assess the efficacy and safety of denosumab, compared with zoledronic acid, for the prevention of SREs in patients with newly diagnosed multiple myeloma. They concluded that denosumab was non-inferior to zoledronic acid, in regards of time to SRE.

We read the paper from Raje et al. [1] with great interest, particularly on the safety results of monthly administration of denosumab versus zoledronic acid in myeloma patients. 

They reported that incidence of osteonecrosis of the jaw (ONJ) was not significantly different between the denosumab and zoledronic acid groups (35 [4%] vs. 24 [3%]; *p* = 0.147).

We want to underline some critical aspects, and give readers our comments.

These reported incidences of “adjudicated” ONJ of 4% and 3%, respectively, are higher values than those previously described in three similar pivotal trials involving bone metastatic cancer patients [2,3,4,5], mostly ranging about 1–2%. Raje et al. suggest in the discussion section of their paper that this difference might be related to a longer drug exposure in their study cohorts [1]. However, we analyzed the data from previous trials on monthly administration of zoledronic acid and denosumab in solid cancers [2,3,4,5,6], and we found similar drug exposure and ONJ time to onset (TTO) for the breast cancer cohort, and a little lower exposure in the other two twin trials. We collected for readers’ convenience the drug exposure data in a table (see Table 1), showing not large differences.

The study protocol recommended (see the Methods section in Raje et al.’s study [1]) that oral examinations were performed at enrollment (non-healed dental or oral surgery was a key exclusion criteria) and every six months thereafter. Furthermore, antiresorptive medication discontinuation was recommended (mandatory after August 2015 amendment) [1], 30 days before an elective invasive oral or dental procedure and until complete mucosal healing occurred. In spite of this careful pre-therapy patient selection and management strategy, invasive dental procedures were reported as the main risk factor in 19 out 35 (denosumab group) and in 13 out of 24 (zoledronic acid group) adjudicated ONJ. It would be interesting to know how many patients received dental procedures overall (i.e., the global treated population), and their reasons. To know the reasons of tooth extractions could be of great value. For example, removal of an unexplainably mobile tooth might be not the risk factor for ONJ, but the trigger of bone exposure of an underlying ONJ disease, undetectable without adequate imaging tools (such as computed tomography).

The myeloma patients were recruited between May 2012 and March 2016, and 59 ONJ cases were “adjudicated” after a median of 17.3 and 13.6 months in the two arms. The definition of ONJ is controversial [7]. The one adopted by Authors seemingly refers to that proposed on 2009 by the Task Force of The American Association of Oral Maxillofacial Surgeons (AAOMS), based on the clinical observation of bone exposure lasting at least eight weeks [8]. Indeed, this definition was revised in 2014 by the same authors, as included cases without bone exposure but only if bone can be probed through a fistula [9]. Non-exposed ONJ (including these latter cases but not limited to them) account for up to 24% of ONJ patients in the literature [7,10,11] and were likely to be overlooked in the present trial. It would be worth to know the influence of this revised definition on the ONJ adjudication process throughout the study, if any.

It would be also relevant to know the number of “potential” ONJ cases registered by investigators, and defined by the presence of clinical sign and symptoms suggestive of ONJ, in the two arms. The rate of “potential” ONJ cases could consequently compared with that one registered in previous solid tumors trials, where only 1/3 of the potential ONJ cases were adjudicated (i.e., in solid tumors trials only 89 adjudicated out of 276 potential cases, according to Saad et al.) [6].

Lastly, as ONJ risk increases up to 15.5% with longer treatment schedules and observation intervals [12,13] after zoledronic acid, denosumab or their sequence, long-term ONJ estimates of the myeloma study (including actuarial risk assessment) is awaited with great interest.

In conclusion, the trial by Raje et al. is able to increase our knowledge about several aspects of ONJ in patients receiving antiresorptive agents. Higher levels of details are needed to ascertain the real ONJ risk of myeloma versus cancer patients, the natural history of ONJ, the risk of underestimation due to a restricted definition of ONJ, the change of the ONJ risk when treatment and follow-up time are prolonged. New reports, with detailed data and actuarial evaluations, are expected in order to obtain evaluable comparison for cost-effectiveness of zoledronic acid and denosumab in myeloma patients.

## Figures and Tables

**Table 1:** Median drug exposure time, median on-study time, median time to onset (TTO) and adjudicated osteonecrosis of jaw (ONJ) rate in zoledronic acid (ZA) versus denosumab studies.

	Median (IQR) Drug Exposure Time		Median (IQR) On-Study Time		Onj Median (IQR) TTO *		Adjudicated ONJ (%)	
	ZA Arm	Denosumab Arm	ZA Arm	Denosumab Arm	ZA Arm	Denosumab Arm	ZA Arm	Denosumab Arm
**Myeloma** (Raje, *Lancet Oncol.* 2018)	14.8 months (7.5–24.9)	15.8 months (8.2–25.8)	17.6 months (9.4–28.1)	17.3 months (8.9–28.5)	13.6 months (8.1–20.3)	17.3 months (7.8–20.9)	3.0%	4.0%
**Breast cancer** (Stopeck, *J. Clin. Oncol.* 2010, 2011)	nr	nr	17 months	17 months	nr ^(1)^	nr ^(1)^	1.4%	2.0%
**Prostate cancer** (Fizazi, *Lancet* 2011)	10.2 months (4.9–16.6)	11.9 months (5.6–18.2)	11.2 months (5.6–17.4)	12.2 months (5.9–18.5)	nr	nr	1.0%	2.0%
**Solid tumors excluding breast and prostate** (including 180 myeloma cases) (Henry, *J. Clin. Oncol.* 2011)	nr ^(2)^	nr ^(2)^	7 months (3–14)	7 months (3–14)	nr	nr	1.3%	1.1%
**All solid tumors** (*Saad. Ann. Oncol.* 2012)	nr ^(3)^	nr ^(3)^	12.1 months (5.4–19.4)	12.1 months (5.6–19.4)	14 months	14 months	1.3%	1.8%

* TTO: time to onset. nr = not reported. IQR: Interquartile Range. ^(1)^ Median number (IQR) of active doses received by the subjects who developed ONJ: 16.5 (8–21) for zoledronic acid and 15 (9.5–19) for denosumab. ^(2)^ Median number (IQR) of active doses received was 7.0 (4–14) for zoledronic acid and 7.0 (4–15) for denosumab. ^(3)^ Median (IQR) number of active doses received was 11.0 (5–19) for zoledronic acid and 13.0 (6–20) for denosumab.
